# Systemic Resistance Induced by Volatile Organic Compounds Emitted by Plant Growth-Promoting Fungi in *Arabidopsis thaliana*

**DOI:** 10.1371/journal.pone.0086882

**Published:** 2014-01-27

**Authors:** Hushna Ara Naznin, Daigo Kiyohara, Minako Kimura, Mitsuo Miyazawa, Masafumi Shimizu, Mitsuro Hyakumachi

**Affiliations:** 1 The United Graduate School of Agricultural Sciences, Gifu University, Gifu City, Japan; 2 Laboratory of Plant Pathology, Faculty of Applied Biological Sciences, Gifu University, Gifu City, Japan; 3 Department of Applied Chemistry, Faculty of Science and Engineering, Kinki University, Higashiosaka-shi, Osaka, Japan; University of Wisconsin-Milwaukee, United States of America

## Abstract

Volatile organic compounds (VOC) were extracted and identified from plant growth-promoting fungi (PGPF), *Phoma* sp., *Cladosporium* sp. and *Ampelomyces* sp., using gas chromatography–mass spectrometry (GC-MS). Among the three VOC extracted, two VOC blends (emitted from *Ampelomyces* sp. and *Cladosporium* sp.) significantly reduced disease severity in *Arabidopsis* plants against *Pseudomonas syringae* pv. *tomato* DC3000 (*Pst*). Subsequently, *m*-cresol and methyl benzoate (MeBA) were identified as major active volatile compounds from *Ampelomyces sp.* and *Cladosporium sp*., respectively, and found to elicit induced systemic resistance (ISR) against the pathogen. Molecular signaling for disease suppression by the VOC were investigated by treating different mutants and transgenic Arabidopsis plants impaired in salicylic acid (SA) or Jasmonic acid (JA)/ethylene (ET) signaling pathways with *m*-cresol and MeBA followed by challenge inoculation with *Pst*. Results show that the level of protection was significantly lower when JA/ET-impaired mutants were treated with MeBA, and in SA-, and JA/ET-disrupted mutants after *m*-cresol treatment, indicating the involvement of these signal transduction pathways in the ISR primed by the volatiles. Analysis of defense-related genes by real-time qRT-PCR showed that both the SA-and JA-signaling pathways combine in the *m*-cresol signaling of ISR, whereas MeBA is mainly involved in the JA-signaling pathway with partial recruitment of SA-signals. The ET-signaling pathway was not employed in ISR by the volatiles. Therefore, this study identified two novel volatile components capable of eliciting ISR that may be promising candidates in biological control strategy to protect plants from diseases.

## Introduction

Non-pathogenic, filamentous, saprophytic rhizosphere fungi that significantly enhance the growth of plants are known as plant growth-promoting fungi (PGPF) [1], [2]. In the search for alternate disease control strategies to minimize the use of chemical pesticides, the discovery of PGPF brought new expectations to researchers worldwide. In the past few years, PGPF from the genera of *Fusarium*, *Penicillium*, *Phoma*, and *Trichoderma* have been frequently studied and evaluated for their high suppressive abilities against a variety of plant diseases as a result of direct antagonism against soil-borne pathogens or by inducing systemic resistance in the plant [3]–[7]. PGPF have been extensively studied to elucidate the mechanisms underlying the disease suppressiveness using different forms of inocula such as barley grain inocula or cell free culture filtrates [6]–[9]. Molecular characterizations of the mechanism of the disease suppressive effects of PGPF or its culture filtrate proved that multiple signaling pathways are involved in ISR by PGPF and are mainly mediated by SA/JA-ET signals [6], [7], [10].

Recent studies have also revealed that volatile organic compounds (VOC) released from some PGPF strains can effectively promote plant growth and enhance disease resistance [11], [12]. In our previous study, we screened about 100 fungal strains by growing them in sealed I-plates (containing a center partition) with tobacco seedlings but without physical contact between the strain and seedling; most plants increased growth when exposed to the volatile substances of the fungi. The volatile blends isolated from *Phoma* sp. GS8-3 significantly increased plant growth at low concentrations [12]. Yamagiwa et al. [11] reported that the volatile compound β-caryophyllene emitted from the PGPF *Talaromyces wortmannii* FS2 significantly enhanced the growth of komatsuna (*Brassica campestris* L. var. *perviridis*) seedlings and their resistance to *Colletotrichum higginsianum*. Although reports on VOC from PGPF are relatively recent and few in number, the role of volatiles emitted from plants and other microorganisms on plant development have been studied extensively [13], [14].

Many reports have focused on the effects of volatiles produced by rhizobacteria or plant growth promoting rhizobacteria on plant disease control. Several volatiles produced by rhizobacteria have exhibited antibacterial or antifungal activities [15]. Two volatiles, 2,3-butanediol and acetoin (3-hydroxy-2 butanone), produced by *Bacillus subtilis* and *Bacillus amyloliquefaciens* have been identified as important factors in inducing systemic resistance and promoting plant growth [14], [16]. Volatiles produced by a few strains of *Streptomyces* are also reported to have potential for biocontrol [17], [18].

While most studies have focused on the interaction between rhizobacteria and plant pathogens, little is known about the plant response to VOC emitted by PGPF and the resistance that is conferred. Therefore, in the present study, we aimed to establish whether the PGPF-released VOC can induce systemic resistance in plants, and if they can, to determine what types of signaling pathways are involved in this ISR. We isolated the VOC from different PGPF and examined the disease suppression efficacy of VOC in a hydroponic culture system using the model plant *Arabidopsis thaliana* (Arabidopsis) and bacterial leaf speck pathogen *Pseudomonas syringae* pv. *tomato* DC3000 (*Pst*) and explicated the molecular basis of VOC-induced ISR in Arabidopsis.

## Materials and Methods

### PGPF Isolates

Fungal isolates *Cladosporium* sp. (D-c-4), *Ampelomyces* sp. (F-a-3) and *Phoma* sp. (GS8-3) used for VOC analysis were collected and identified at the laboratory of Plant Pathology, Gifu Univerisity.

### Test Plants and Pathogen

Seeds of *Arabidopsis thaliana* ecotype Columbia (Col-0) were provided by Dr. K.S. Park (NIAST, Suwon, Korea). Mutants *ein3* [19], *npr1* [20] and *jar1* [21] were obtained from NASC (The Nottingham *Arabidopsis* Stock Centre) and transgenic line NahG was a personal gift [22]. All the mutants and transgenic *Arabidopsis* lines were developed against the background of the Col-0 ecotype. Virulent pathogen *Pseudomonas syringae* pv. *tomato* (*pst*) DC3000 was provided by Y. Ichinose (Okayama University, Okayama, Japan).

### Extraction and Analysis of Volatile Metabolites from PGPF Isolates

Three PGPF isolates were cultured in 10 mL solid phase micro extraction (SPME) vials (Supelco, Sigma-Aldrich Co. US), and the volatile metabolites were extracted by headspace SPME during 30 min at 25°C. Polydimethylsiloxane/divinylbenzene (PDMS/DVB) (65 µm) fibers were used for volatile profiling. Fibers were obtained from Supelco and conditioned before analyses according to the manufacturer’s recommendations. The composition of volatile organic compounds, isolated from *Phoma* sp. (GS8-3), *Ampelomyces* sp. (F-a-3) and *Cladosporium* sp. (D-c-4), were identified using GC-MS analysis as described by Miyazawa et al. [23]. Compounds were identified using the U.S. National Institute of Standards and Technology (NIST) Mass Spectral Library or by comparing the retention times and spectra with those of authentic standards and Kovats retention indices with literature data.

### Hydroponic Culture of Plants

Arabidopsis plants were grown in a hydroponic culture system developed by Toda et al. [24]. In this system, seeds were sown on nylon mesh (50 holes per inch) and were placed in a plastic photo-slide mount (50×50 mm; Fuji film, Japan). These mesh mounts were floated in a plastic case with the help of small pieces of styrofoam on 5 L of 1∶10 MGRL nutrient solution (pH 5.6) and kept in a growth chamber at 24°C with a 12 h day/12 h night cycle [25]. The nutrient solution was renewed every 7 days, and the culture was continued for 2 weeks.

### Application of Volatile Organic Compounds (VOC)

The volatile compounds, isolated from *Phoma* sp. (GS8-3), *Ampelomyces* sp. (F-a-3) and *Cladosporium* sp. (D-c-4) (Table 1) that were identified through GC-MS analysis and commercial methacrylic acid and isobutyl acetate (synthetic chemicals) were dissolved in CH_2_Cl_2_ and diluted to a 0.1 M solution. VOC were mixed with 0.1 g of lanolin before use and then 50 µL of one of the VOC was applied to a sterile paper disk and kept on a glass petri dish (3 cm). A dilution series (1 µM to 100 mM) of *m*-cresol and MeBA was also prepared and used to analyze dose-specific effects on disease severity. Hydroponically grown, 13-d-old *Arabidopsis* plants were transferred to a medium-sized (13×32×18.5 cm) plastic case containing 1/10 MGRL and kept in a large plastic case with the VOC in the glass petri dish. The whole system was then covered quickly and held for 24 h before inoculation with the pathogen.

**Table 1:** Retention index (RI) and peak areas for volatile organic compounds (VOC) extracted from 14-d-old cultures of the plant-growth-promoting fungi *Phoma* sp. (GS8-3), *Ampelomyces* sp. (F-a-3) and *Cladosporium* sp. (D-c-4) using SPME-based GC-MS analysis.

		Peak areas (%)		
Compounds	RI	*Phoma* sp. GS8-3	*Ampelomyces sp*. F-a-3	*Cladosporium* sp. D-c-4
2-Methyl-propanol		9.4	3.0	–
3-Methyl-butanol		83.8	22.6	–
4-Heptanone		–	2.5	–
2-Heptanone		0.4	–	–
2-Heptanol		0.4	–	–
3-Octanone	986	–	1.1	–
*m*-Methyl-anisole	1022	–	1.9	–
4-Methyl-phenol	1080	3.3	–	–
*m*-Cresol	1081	–	59.8	–
Methyl benzoate	1095	–	–	100.0
Phenylethyl alcohol	1116	2.8	8.6	–
Cubenene	1376	–	0.6	–
Total		100.0	100.0	100.0

Note: Compounds were identified by comparing the RI and mass spectra with data in the NIST database.

### Inoculation

The virulent bacterium *Pst* DC3000 was cultured in Kings’ B broth containing rifampicin (50 mg/L) for 2 days at 28°C. The bacterial cells were collected by centrifugation, washed twice with sterilized distilled water (SDW) and resuspended in SDW to a final concentration of 7.0×10^7^–8.0×10^7^ colony forming units (cfu)/mL (OD_600_ = 0.070–0.080). The surfactant Silwet L-77 (0.01% v/v; Nihon Unica, Tokyo, Japan) was added as a spreading agent during inoculation. One day after the VOC treatment, 2-wk-old plants were sprayed with 200 mL of bacterial suspension. The inoculated plants were then kept at 100% relative humidity in the dark for 2 days to induce disease development. Plants were then transferred to the growth chamber with 12 h day/12 h night cycle and held for 3 more days.

### Assessment of Disease Severity

Five days after the pathogen challenge, disease severity was scored, and the number of colony forming units of *Pst* (cfu)/g of leaves was determined for 10 randomly selected plants. Severity was scored for each plant as the percentage of total leaf surface with symptoms, from 0 = no symptoms to 100 = most severe with necrotic symptoms, and calculated using the formula described by Hossain et al. [6]. To determine the number of *Pst* DC3000 cells in inoculated leaves, we collected and weighed all leaves from the samples, rinsed them thoroughly in sterile water, then homogenized them in sterilized distilled water. Leaf suspensions were plated on KB agar supplemented with rifampicin (50 mg/L), and after 48 h incubation at 28°C, the number of cfu of *Pst* per gram of leaves was calculated. The experiment was repeated 3 times.

### RT-PCR Analysis

After the 24-h VOC treatment, aerial parts from 15 randomly selected plants were sampled in 1.5 mL Eppendorf tubes, ground in liquid nitrogen and homogenized with 600 µL of the extraction buffer (20 g of guanidine thiocyanate, 0.2 g of *N*-lauroylsarcosine sodium salt and 0.2 g of trisodium citrate dihydrate dissolved in 40 mL of RNase free water) and 10 µL of 2-mercaptoethanol. The aqueous phase resulting from centrifugation at room temperature was re-extracted with a phenol : chloroform : isoamyl alcohol (PCI) (25∶ 24∶ 1; v/v) mixture. The upper aqueous phase was precipitated with isopropanol followed by a 75% ethanol rinse. The precipitated RNA was collected, air-dried briefly and dissolved in RNase-free water. After treatment with RNase-free DNase and inactivation of the DNase according to the instructions of the supplier (Takara Bio, Shiga, Japan), approximately 1 µg of total RNA was reverse transcribed to single-strand cDNA, and a sample of the obtained cDNA was amplified by RT-PCR, as described by Suzuki et al. [26] to analyze the expression of a set of well-characterized defense-related genes. The expression of candidate priming gene was analyzed using the following primers: F-5^′^-GTAGGTGCTCTTGTTCTTCC-3^′^, R- 5^′^-TTCACATAATTCCCACGAGG-3^′^ (*PR-1;*At2G14610, product size 421 bp) and F-5^′^-AATGAGCTCTCATGGCTAAGTTTGCTTCC-3^′^), R-5^′^-AATCCATGGAATACACACGATTTAGCACC-3^′^ (*PDF*1.2a; At5G44420, product size 281 bp). Expression of defense-related genes was determined by semi-quantitative RT-PCR. PCR products were separated on a 1.5% agarose gel, and intensities of bands were scanned with Typhoon 9400 Variable Mode Imager (GE Healthcare UK, Amersham, UK). The signal strength of each band was expressed numerically with the program image Quant 5.2 (GE Healthcare), and the relative expression level of each gene was calculated. β-tublin (*TUB8*; AT5G23860) was used as an internal standard using primers Forward-5^′^-CGTGGATCACAGCAATACA-3^′^ and Reverse-5^′^-CCTCCTGCACTTCCACTT-3^′^.

### Real-time Quantitative RT-PCR Analysis

Real-time RT-PCR assay was performed using real-time PCR, ABI PRISM 7000 system (Applied Biosystems, Tokyo, Japan) using the default thermocycler program for all genes. Approximately 1 µg of total RNA was reverse transcribed to single-strand cDNA as described by Suzuki et al. [26] after inactivation of DNase I according to the manufacturer’s instructions (Takara Bio, Shiga, Japan). A sample of the obtained cDNA was amplified to monitor the expression of a set of selected genes. Power SYBR Green Master Mix was used according to the manufacturer’s instruction; 1 µL of cDNA to 10 µL of SYBR Green Master mix: 0.8 µL of 5 µM primer F&R: 7.4 µL SDW. Primers used for real-time PCR are listed in Table 2. The relative signal intensity compared with control plants was calculated using 2^−ΔΔCt^ from the threshold cycle (Ct) values according to the manufacturer’s software. Relative RNA levels were calibrated and normalized against expression levels of the internal control genes *UBQ5* and *ACT2*.

**Table 2:** Gene-specific primers used in real-time qRT-PCR analysis.

AGI code	Target gene	Primer sequences	Product size (bp)
Salicylic acid regulated gene			
At2g14610	PR-1	F5^′^-TTCTTCCCTCGAAAGCTCAA-3^′^	174
		R 5^′^-AAGGCCCACCAGAGTGTATG-3^′^	
At3g57260	PR-2	F 5 ^′^-AGCTTAGCCTCACCACCAATGT-3^′^	83
		R 5^′^-CCGATTTGTCCAGCTGTGTG-3^′^	
At1g75040	PR-5	F 5^′^-TGTTCATCACAAGCGGCATT-3^′^	99
		R5^′^GTCCTTGACCGGCGAGAGTTAATGCCGC-3^′^	
Jasmonic acid/Ethylene regulated gene			
At3g12500	PR-3	F 5^′^-GGCCAGACTTCCCATGAAAC-3^′^	113
		R 5^′^-CTTGAAACAGTAGCCCCATGAA-3^′^	
At3g04720	PR-4	F 5^′^-GCAAGTGTTTAAGGGTGAAGAACA-3^′^	104
		R 5^′^-GAACATTGCTACATCCAAATCCAAG-3^′^	
At5g44420	PDF1.2	F 5^′^-TTTGCTGCTTTCGACGCAC-3^′^	80
		F 5^′^-CGCAAACCCCTGACCATG-3^′^	
At5g24770	AtVSP2	F 5^′^-TCAGTGACCGTTGGAAGTTGTG-3^′^	104
		R 5^′^-GTTCGAACCATTAGGCTTCAATATG-3^′^	
At1g32460	MYC2	F 5^′^-AGCAACGTTTACAAGCTTTGATTG-3^′^	76
		R 5^′^-TCATACGACGGTTGCCAGAA-3^′^	
Housekeeping gene/internal control			
At3g62250	UBQ5	F 5^′^-GACGCTTCATCTCGTCC-3^′^	256
		R 5^′^-GTAAACGTAGGTGAGTCCA-3^′^	
At2g37620	ACT2	F 5^′^-AGTGGTCGTACAACCGGTATTGT-3^′^	92
		R 5^′^-GATGGCATGAGGAAGAGAGAAAC-3^′^	

### Statistical Analysis

The experimental design was completely randomized, consisting of three replications for all treatments. The experiment was repeated at least twice. Data were subjected to analysis of variance (ANOVA). A Student’s *t*-test and Bonferroni multiple comparison test were used to determine statistically significant differences between treated samples and untreated control.

## Results

### Extraction and Identification of Volatile Metabolites from PGPF Isolates

When the volatile metabolites were extracted from 2-wk-old cultures of three PGPF isolates using headspace SPME and identified using gas chromatography–mass spectrometry (GC-MS), most of the VOC from *Phoma* sp. (isolate GS8-3) and *Ampelomyces* sp. (isolate F-a-3) were C4–C8 hydrocarbons (Table 1). Volatiles from *Phoma* sp. (GS8-3) comprised 2-methyl-propanol (9.4%), 3-methyl-butanol (83.8%), 2-heptanone (0.4%), 2-heptanol (0.4%), 4-methyl-phenol (3.3%) and phenylethyl alcohol (2.8%). Whereas, VOC from *Ampelomyces* sp. (F-a-3) comprised 2-methyl-propanol (3%), 3-methyl-butanol (22.6%), 4-heptanone (2.5%), 3-octanone (1.1%), *m*-methyl-anisole (1.9%), *m*-cresol (59.8%), phenylethyl alcohol (8.6%) and cubenene (0.6%). Only one volatile component, methyl benzoate (MeBA) (100%), was identified from *Cladosporium* sp. (isolate D-c-4 ).

### VOC Emitted from PGPFs Suppress Disease Severity

**Figure 1:**
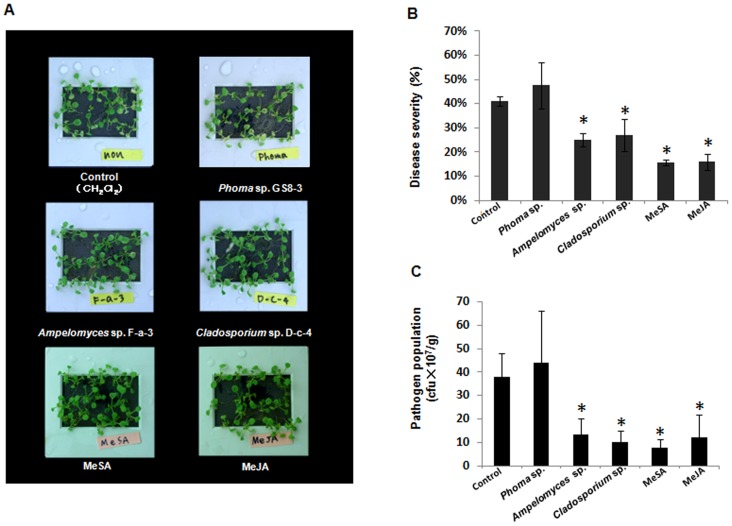
Suppression of disease symptoms and numbers of *Pst* DC3000 after VOC pretreatment in *Arabidopsis thaliana*. A. Plants (17-d-old) on mesh screen in a slide mount 5 days after challenge inoculation with *Pst* DC3000. Plants were treated with volatile compounds emitted by *Phoma* sp. GS8-3, *Ampelomyces* sp. F-a-3 and *Cladosporium* sp. D-c-4 for 24 h then inoculated with *Pst*. Volatiles emitted from *Phoma* sp. and *Ampelomyces* sp. were used as blend of volatiles and from *Cladosporium* sp. was methyl benzoate (MeBA) only. Control was treated with CH_2_Cl_2_ only; MeSA and MeJA were used as positive controls. B. VOC-induced reduction of disease severity. Severity was scored for each plant as the percentage of total leaf surface with symptoms, from 0 = no symptoms to 100 = most severe with necrotic symptoms. C. Growth of *Pst* DC3000 (cfu g^−1^ fresh mass) in leaves. Asterisks indicate values differ significantly (Student’s *t*-test, *P* = 0.01) from the control. Data are from representative experiments that were repeated at least 3 times with similar results.

Arabidopsis plants were treated with one of the volatile organic compounds isolated from the 3 PGPFs (*Phoma* sp., *Ampelomyces* sp. and *Cladosporium* sp.) in hydroponic culture (Table 1). After 24 h of treatment, plants were inoculated with bacterial leaf speck pathogen *P. syringae* pv. tomato (*Pst*) DC3000, and disease symptoms and number of bacteria were evaluated 5 days after inoculation. As shown in Fig. 1 (A, B), Arabidopsis Col-0 plants treated with the VOC isolated from *Ampelomyces* sp. F-a-3 and *Cladosporium* sp. D-c-4 resulted in a significant reduction in disease severity compared with the control. Disease severity, based on an index for percentage of total leaf surface with symptoms then calculated as the percentage protection compared with the control, in Arabidopsis plants was 39% after treatment with VOC from *Ampelomyces* sp. F-a-3 and 34% after treatment with VOC isolated from *Cladosporium* sp. D-c-4 (MeBA). On the other hand, disease severity in plants treated with VOC isolated from *Phoma* sp. (GS8-3) was higher than in the control. Results in Fig. 1(C) present the number of colony-forming units (cfu g^−1^) of *P. syringae* pv. tomato (*Pst*) DC 3000 in challenged leaves and reveal that the plants treated with VOC from *Ampelomyces* sp. F-a-3 and *Cladosporium* sp. D-c-4 caused an approximately 2.4- and 3.8-fold decrease in cfu g^−1^, respectively, compared with the control.

### VOC Induced High Expression of Defense-related Genes

**Figure 2:**
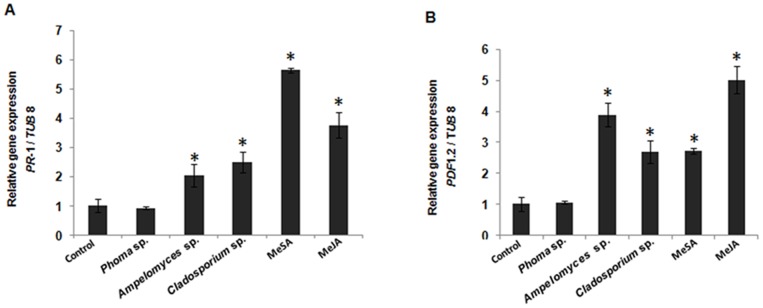
Expression of of defense-related genes. A. SA-responsive gene *PR*-1 and B. JA-responsive gene *PDF* 1.2 in leaves of *Arabidopsis thaliana* treated with volatile blends emitted from *Phoma* sp. and *Ampelomyces* sp. and MeBA from *Cladosporium* sp. in semi-quantitative RT-PCR analysis. Asterisks indicate statistically significant differences (Student’s *t*-test, *P* = 0.01) compared with the control. Data are from representative experiments that were repeated at least 3 times with similar results.

To evaluate the roles of SA and JA in the VOC-induced defense responses in Arabdiposis, the expression of SA- and JA-dependent marker genes was analysed by semi-quantitative PCR (Fig. 2. A and B). The expression level of the SA-inducible gene *PR-*1 and of JA-inducible gene *PDF* 1.2 was significantly higher in aerial parts of Arabidopsis treated with VOC isolated from *Ampelomyces* sp. F-a-3 and *Cladosporium* sp. D-c-4 (MeBA) than in the control. On the other hand, *Phoma* sp. (GS8-3) emitted VOC-treated plants did not express defense-responsive genes. Expression of *PR*-1 was 2 and 2.5 times higher than in the control in *Ampelomyces* sp. F-a-3 and *Cladosporium* sp. D-c-4 emitted VOC-treated plants, respectively. *PDF* 1.2 was expressed 3.9 and 2.6 times higher in *Ampelomyces* sp. and *Cladosporium* sp. emitted VOC-treated plants, respectively, over the control. Thus, both SA- and JA-signalling are involved in the VOC-induced defence in Arabidopsis.

Because MeBA was identified as the major (100%) volatile compound emitted by *Cladosporium* sp. D-c-4 that elicits ISR (Figs. 1, 2), while VOC from *Ampelomyces* sp. F-a-3 was extracted as a blend of volatiles (Table 1), we further analyzed the VOC isolated from *Ampelomyces* sp. F-a-3 to identify the major active volatile compound emitted by that.

### m-Cresol is a Major Component with an Important Role in Disease Supression by *Ampelomyces* sp. (F-a-3)

**Figure 3:**
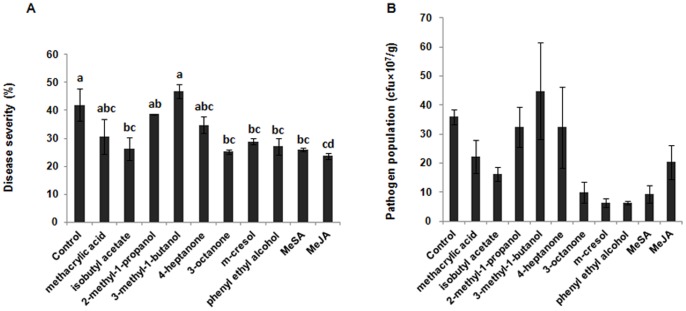
Supression of disease symptoms and pathogen population by VOC isolated from *Ampelomyces* sp. A. VOC-induced reduction of disease severity caused by *Pst* DC3000 in Arabidopsis. Severity was scored for each plant as the percentage of total leaf surface with symptoms, from 0 = no symptoms to 100 = most severe with necrotic symptoms. B. Growth of *Pst* DC3000 (cfu g^−1^ fresh mass) in leaves. Plants were pretreated with 50 µL of one of the volatile components (0.1 M) for 24 h before inoculation. Methacrylic acid and isobutyl acetate were also tested as volatiles. Controls received only CH_2_Cl_2_; MeSA and MeJA were used as positive control treatments. Different letters indicate significant differences between treatments according to Bonferroni multiple comparison test (P = 0.05). Data are from representative experiments that were repeated at least 3 times with similar results.

As we see in Figs. 1 and 2, blend of volatiles isolated from *Ampelomyces* sp. and MeBA isolated from *Cladosporium* sp. significantly suppressed disease against *Pst* DC3000. In the blend of volatiles produced by *Ampelomyces* sp. F-a-3, *m*-cresol occupied the leading position (59.8%). Therefore, in the next step, we analysed all the components extracted from *Ampelomyces* sp. for their ability to reduce disease and the pathogen population. Together with the F-a-3 volatiles, 2 of the VOC, methacrylic acid and isobutyl acetate, found to be common components in 3- and 5-d-old cultures of *Phoma* sp. GS8-3 in our previous study [12], were also included in the ISR test. Fig. 3 shows that 3 of the VOC from *Ampelomyces* sp. (F-a-3), 3-octanone, *m*-cresol, phenyl ethyl alcohol, and the test volatiles methacrylic acid and isobutyl acetate induced systemic resistance in Arabidopsis against *Pst* DC3000 by 5 days after inoculation. Among the VOC, 3-octanone was highly effective in disease supression, and the bacterial population, although there was no sifnificance differences between treatments and control, was reduced the most by treatment with *m*-cresol. From 14-d cultures, 3-octanone was identified as a trace component (1.1%), whereas *m*-cresol was greatest (59.8%) in the total volatile blend. We thus considered this compound to be the major active volatile component involved in the ISR by *Ampelomyces* sp. F-a-3.

### Dose-specific Effects of *m*-cresol and MeBA on ISR

**Figure 4:**
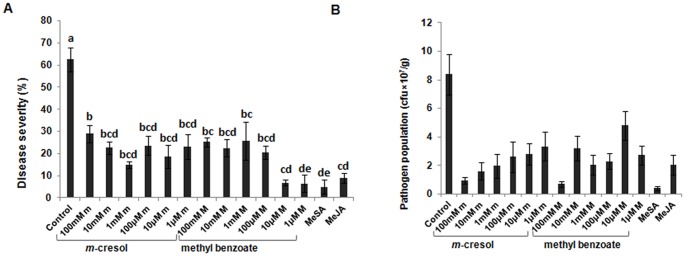
Systemic resistance induced by *m*-cresol and methyl benzoate (MeBA) at different concentrations. A. Reduction in disease severity and B. Growth of *Pst* DC3000 in leaves after pretreatment of plants with *m*-cresol and MeBA at different concentrations followed by challenge inoculation with *Pst* DC3000. Disease severity was scored for each plant as the percentage of total leaf surface with symptoms, from 0 = no symptoms to 100 = most severe, with necrotic symptoms. Different letters indicate significant differences between treatments according to Bonferroni multiple comparison test (P = 0.05). Data are from representative experiments that were repeated at least 3 times with similar results.

To observe the effects of *m*-creosl and MeBA on ISR at different concentrations, we pretreated plants with a dilution series of the compounds (1 µM to 100 mM) before pathogen inoculation, then scored the percentage disease severity and the pathogen population. As we see in Fig. 4, both *m*-cresol and MeBA induced ISR at all concentrations although the effect varied at different concentrations of *m*-cresol and MeBA. The pathogen population was decreased the most at 100 mM. However, *m*-cresol and MeBA both induced ISR significantly over the control even at low concentrations.

### Systemic Resistance Induced by Methyl Benzoate is Compromised in Arabidopsis Genotypes Defective in JA-dependent Signalling Pathway

**Figure 5:**
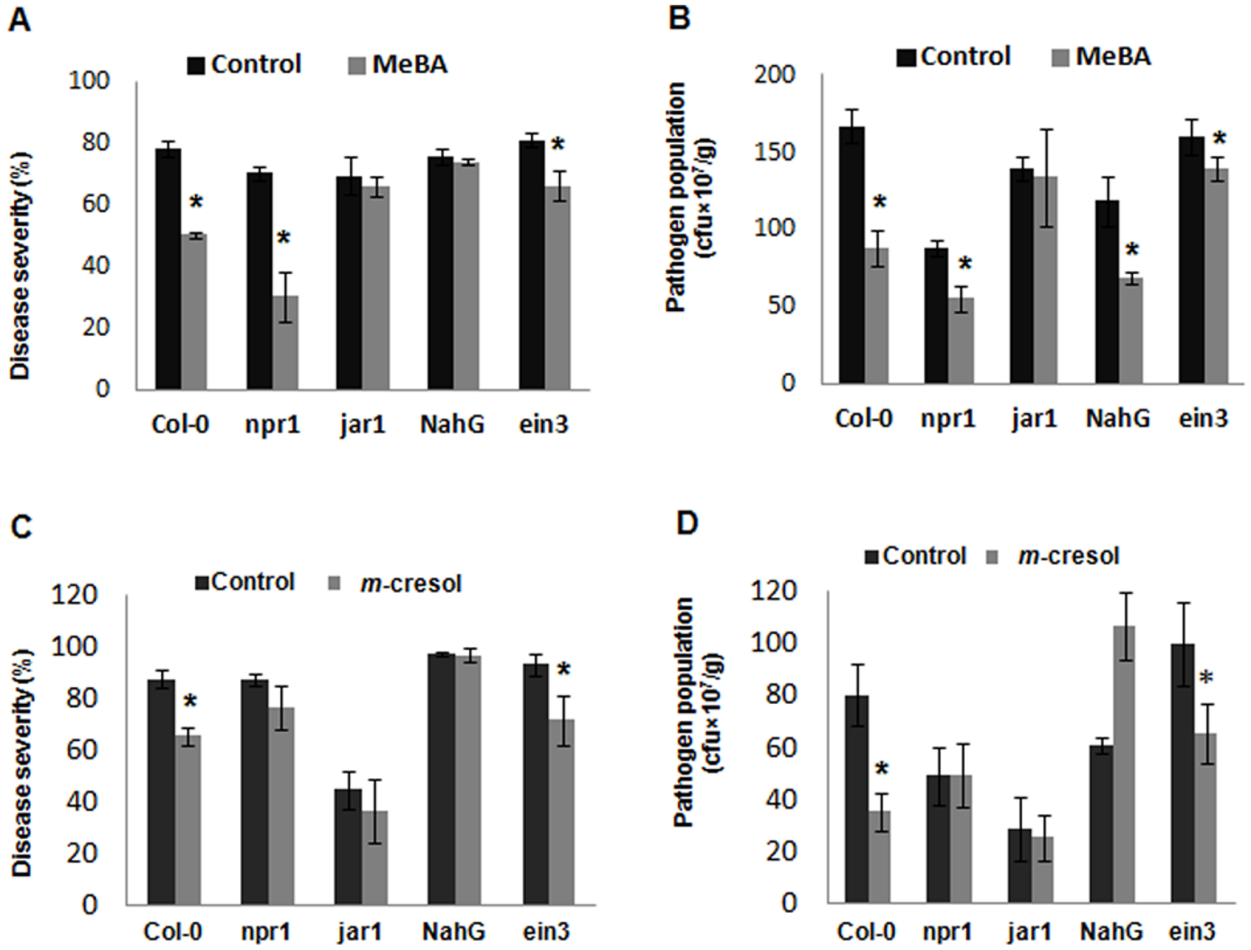
Suppression of disease symptoms and *Pst* DC3000 population by VOC methyl benzoate (MeBA) and *m*-cresol. Arabidopsis transgenic plants and mutants impaired in defense signalling pathways and wild-type (Col-0) plants were used. A. Reduction in disease severity and B. Growth of *Pst* DC3000 in leaves after MeBA pretreatment followed by challenge inoculation with *Pst* DC3000. C. Reduction in disease severity and D. Growth of *Pst* DC3000 in leaves after *m*-cresol pretreatment followed by challenge inoculation with *Pst* DC3000. Data are percentage of disease severity (scored for each plant as the percentage of total leaf surface with symptoms, from 0 = no symptoms to 100 = most severe with necrotic symptoms) or number of cfu g^–1^ fresh mass 5 days after challenge inoculation. Asterisks indicate statistically significant differences (Student’s *t*-test, *P* = 0.01) compared with the control. Data are from representative experiments that were repeated at least 3 times with similar results.

Previously, we checked the induction of defense-related genes *PR*-1 and *PDF*-1.2 in Arabidopsis plants treated with VOC blends (Fig. 2). To elucidate the signalling pathways leading to the ISR mediated by the major VOC, we exposed different Arabidopsis mutants or transgenic plants that are impaired in a specific regulatory pathway to the major VOC that triggered ISR: SA-deficient mutant *npr1*, impaired in NPR1 activity or nonexpressor of PR genes; Arabidopsis transgenic plant NahG, defective in SA-dependent signalling; an ethylene-insensitive 3 (*ein*3) mutant and a JA-deficient mutant *jar1.* Application of methyl benzoate (MeBA) extracted from *Cladosporium* sp. D-c-4 significantly decreased development of leaf specks caused by *Pst* DC3000 in the *npr1* mutant, impaired in NPR1 activity and in the *ein3* mutant, impaired in ET-dependent signalling (Fig. 5 A). Bacterial growth also followed a trend similar to lesion development in *npr1* and *ein3* (Fig. 5 B), indicating that ISR mediated by MeBA is independent of SA and ET signalling. On the other hand, disease severity and the pathogen population were higher in the JA-signalling defective *jar1* mutant implicating the involvement of JA-signalling pathways in ISR by MeBA. Remarkably, disease severity in Arabidopsis transgenic plant NahG was not signficantly reduced by treatment with MeBA, albeit the bacterial population was significantly lower than in the control. This result indicates a partial recriutment of the signal transduction molecule SA in MeBA-mediated ISR.

### *m*-Cresol Failed to Induce Systemic Resistance in Arabidopsis Mutants Impaired in SA−/JA-dependent Signalling Pathways

*m*-Cresol was also tested to determine the molecular patterns of induced systemic resistance in Arabidopsis plants using the same set of genotypes as those used in the MeBA treatment. Results showed that the percentage protection and the reduction of bacterial population were compromised in the SA-signalling-defective transgenic plant NahG, the NPR1-activity-impaired mutant *npr1*, and the JA- signalling-impaired mutants *jar1* plants treated with *m*-cresol (Fig. 5. C&D). On the other hand, lesion development and proliferation of bacterial pathogens in ET-signalling-impaired Arabidopsis mutant plants were significantly reduced in contrast to the control. These results indicate that the SA-signalling pathway is essential for *m*-cresol-induced systemic resistance in Arabidopsis plants, including partial JA-signalling.

### Induction of Arabidopsis Defense-related Genes in Plants Treated with Major VOC, MeBA and *m*-cresol

**Figure 6:**
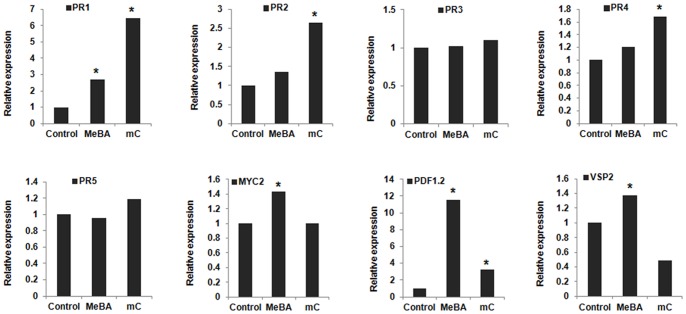
Relative expression of defense-related genes on leaves of *A. thaliana* treated with *m*-cresol and MeBA. Amplification of JA−/ET-responsive genes *PR3* and *PDF1.2*, JA-responsive genes *AtVSP2* and *MYC2*, ET-responsive gene *PR4*, and SA-inducible genes *PR1*, *PR2* and *PR5* were analyzed with real-time qRT-PCR. Leaves from 15 representative plants were sampled 5 days after inoculation. Asterisks indicate statistically significant differences (Student’s *t*-test, *P* = 0.01) compared with the control treatment.

To define more clearly the role of SA-, JA- and ET-signal transduction pathways in the induction of systemic resistance by VOC, we further studied the induction pattern of marker genes for these pathways in plants exposed to the major VOC (Table 2). Plants were treated with VOC for 24 h, and transcription of SA-inducible gene *PR1, PR2, PR5* and ET-inducible gene *PR4*, JA−/ET-inducible gene *PR3, PDF1.2* and JA-inducible *AtVSP2* and *MYC2* was analysed by real-time quantitative RT-PCR. Result showed that relative expression of SA-inducible gene *PR1* and *PR2* was significantly higher (more than 6-fold and 2.5-fold, respectively) in *m*-cresol-treated plants (Fig. 6). *PR1* also showed high expression after the MeBA treatment (>2 fold), supporting the previous data on SA involvement (Fig. 2). On the other hand, JA/ET-inducible marker gene *PDF1.2*, JA-inducible gene *MYC2*, and *VSP2* showed significantly higher relative expression in case of MeBA-treated plants. *m*-Cresol also significantly induced the JA/ET-inducible gene *PDF1.2* (>2 fold), strengthening support for the involvement of JA based on the previous data (Figs. 2, 5). The expression of ET-inducible marker gene *PR4* was also higher (>1.6 fold) after *m*-cresol treatment, differing from the data in Fig. 5, but not after the MeBA treatment. The JA/ET-inducible gene *PR3* and SA-inducible marker gene *PR5* were not noticeably expressed in our experiments.

## Discussion

In our previous study, we validated that chemical signals were being emitted into the air from the fungi *Phoma* sp. (GS8-3), *Ampelomyces* sp. (F-a-3) and *Cladosporium* sp. (D-c-4) and contributed to promoting the growth of tobacco seedlings [12]. Here, we isolated the VOC from these PGPF and analyzed their potential for plant protection by pretreating Arabidopsis plants and challenging them with the pathogen *Pseudomonas syringae* pv. *tomato* DC3000 (*Pst*). Protection of the plant was manifested by both a reduction in disease severity and a decrease in pathogen proliferation in the leaves. The VOC emitted from the PGPF suppressed *Pst* infection via induced systemic resistance since there was less disease without direct contact between the VOC and the pathogen. *Phoma* sp. (GS8-3) and *Ampelomyces* sp. (F-a-3) emitted a blend of volatile components, whereas only one volatile (MeBA) was produced by *Cladosporium* sp. (D-c-4) after 14 days of culture (Table 1). Different strains of *Phoma* sp. including GS8-3 have previously been reported to promote growth and induce systemic resistance in plants [27], [28]. In addition, we found that a volatile blend emitted by GS8-3 was able to increase plant growth [12]. But unexpectedly, in the present study, the VOC isolated from plants treated with *Phoma* sp. (GS8-3) did not suppress disease or reduce the pathogen population after inoculation with the pathogen (Fig. 1). On the other hand, volatile components isolated from *Ampelomyces* sp. (F-a-3) and *Cladosporium* sp. (D-c-4) did reduce disease symptoms and pathogen population significantly, as did the positive control treated with MeJA and MeSA. The mycoparasite *Ampelomyces quisqualis*, a well-known biocontrol agent, is widely used for controlling powdery mildew of different plants and is known to act by hyperparasitism [29], [30], but our finding that *Ampelomyces* sp. emits VOC that can induce systemic resistance is undisputedly the first report for this antagonist. Likewise, *Cladosporium* spp. is also a mycoparasite of powdery mildew fungi [31], parasitizing the surface of the penicillate cells of the cleistothecia and causing plasmolysis of the conidia [31], [32]. Antifungal compounds were presumed to play role in this inhibitory effect or antibiosis, but the mode of action had not been studied in detail.

In the present study, we isolated a volatile compound from *Cladosporium sp*. (D-c-4) that could induce systemic resistance in plants. In addition, to determine the mode of action underlying the ISR by the VOC extracted from the PGPF strains, we checked two Arabidopsis defense-related genes *PR*-1 (SA) and *PDF*1.2 (JA/ET) for post-inoculation amplification. Our results showed that disease suppression by the VOC isolated from both F-a-3 and D-c-4 involved the SA and JA/ET pathways (Fig. 1 B), with methyl benzoate (C_6_H_5_CO_2_CH_3_) the only compound (100%) emitted by *Cladosporium* sp. (D-c-4). In the mixture of VOC emitted by *Ampelomyces* sp. (F-a-3), *m*-cresol (CH_3_C_6_H_4_OH) significantly induced systemic resistance and was the most abundant of all the VOC, confirming it as the major active volatile compound in ISR (Fig. 3). Methacrylic acid and isobutyl acetate, were isolated as common components from *Phoma* sp. GS8-3 after 3 and 5 days of culture in our previous study [12], so we included them in ISR tests. Because the volatiles varied in number and quantity over time during culture [12], we isolated VOC from a 14-d-old fungal culture, when methacrylic acid and isobutyl acetate are absent from the VOC profile of *Phoma* sp. GS8-3. In Fig. 3, we see the major volatiles emitted from GS8-3; 2-methyl-1-propanol and 3-methyl-1-butanol failed to reduce disease and pathogen population in Arabidopsis. On the contrary, methacrylic acid and isobutyl acetate reduced disease severity and the pathogen population, leaving little doubt that the age of the fungal culture is the likely reason behind the negative effects of VOC emitted by *Phoma* sp.GS8-3 in Arabidopsis; however, we did not test this further. From our results, the volatile compounds, methacrylic acid, isobutyl acetate, 3-octanone, *m*-cresol and phenyl ethyl alcohol, were found to reduce disease severity, and are potential candidates for biological control agents.

When we used these two major volatile organic components and well-characterized mutants and transgenic plants to clarify the signaling pathways involved in this VOC-mediated ISR, our data revealed that plant protection was completely arrested in mutant *jar1* after treatment with MeBA, a paradigm of JA-dependency. Although JA and ET are thought to be the signal transduction molecules for induced systemic resistance (ISR) by biological control agents and JA and ET share a common pathway in ISR [33], in our case, disease in an ethylene-impaired mutant plants (*ein3*) was significantly suppressed, similar to the wild-type plants, indicating that an independent JA-signalling pathway is involved in MeBA-accelareted ISR. Disease severity in Arabidopsis transgenic NahG, defective in SA-dependent signaling, was higher although the pathogen population was significantly reduced compared with the control. But NPR1-activity-impaired mutant plants did not differ from wild-type plants in being protected by the volatile-induced resistance. Previously, PGPF or PGPR (rhizobacteria)- mediated ISR in Arabidopsis was reported to involve a novel signaling pathway based on JA/ET signals and regulated by NPR1 [6], [7], [33,]. But in our case, PGPF-regulated MeBA-triggered ISR signalling pathways appear to be involved, mainly via JA as a signal molecule with the partial recruitment of SA, but the ISR signaled via JA/ET differs by requiring NPR1.

Similar to MeBA, *m*-cresol also induced ISR without involving an ET-signal molecule but involved a JA-signaling pathway. Our results showed that *m*-cresol used a complete SA-dependent signalling pathway to trigger ISR that requires NPR1. The signal transduction pathway through SA accumulation is found in the systemic acquired resistance (SAR) induced by pathogen attack [34], while it is thought that JA and ET are the signal-transducing molecules for induced systemic resistance (ISR) by biocontrol agents (BCAs) [33]. However, there are some reports that SA can also work as an inducement factor of ISR by BCAs [6], [7]. Our results also proved the involvement of both SA- and JA-signal transduction in ISR.

For more confirmation of the molecular mechanisms behind the VOC-mediated ISR, we assessed transcription levels of Arabidopsis defense-related markers, SA-, JA/ET-inducible genes (Table 2) by real-time qRT-PCR analysis. Like the results of the mutant screening, *m*-cresol significantly induced the SA-inducible marker genes *PR1* and *PR2*, confirming that the volatile lowered disease severity by inducing systemic resistance mainly through the SA-signal transduction pathway. In addition, JA-inducible gene *PDF1.2* was expressed significantly by treating plants with *m*-cresol, strengthening our idea of a partial engagement of JA-regulation. On the other hand, of all the genes examined, expression of the JA/ET-signal gene *PDF1.2* was the highest in MeBA-treated plants, more than 11-fold higher than in the control. Moreover, the JA-inducible marker genes *MYC2* and *VSP2* were also amplified significantly by the MeBA treatment. Thus, we are more confident that the JA-signaling pathway is activated in MeBA-mediated ISR in Arabidopsis. MeBA also induced transcription of the SA-responsive *PR1* gene, supporting our mutant-screening data. Generally, regulation of *PDF1.2* after pathogen infection requires concomitant activation of JA- and ET-signaling pathways. However, our results provide substantial evidence that the PGPF-emitted VOC *m*-cresol and MeBA induce *PDF1.2* using the JA-signal independently of ET-signaling. Although, we cannot explain the reason, the ET-responsive gene *PR*4 was significantly expressed by *m*-cresol treatment compared with the control, whereas ET-impaired mutants (*ein2*) showed no involvement of ethylene in the resistance elicited by MeBA or *m*-cresol. However, studies on other volatile components from different sources indicated various modes of action can be involved. For instance, Ryu et al. [14] revealed that the rhizobacterial volatile 2-3-butanediol and acetoin employed an ET-signaling pathway independent of the SA- and JA-signals, completely opposite of the mechanism induced by our volatiles. Lee et al. [35] also found ISR by a long-chain volatile isolated from *Paenibacillus polymyxa* E681 that primed expression of SA-, JA- and ET-signaling marker genes. From another study, C6-aldehyde volatiles from green leaves of Arabidosis induced resistance involving the JA-signaling pathway in Arabidopsis against a necrotrophic pathogen [36]. However, the response of Arabidopsis to different volatile compounds differed because the amount and type of the elicitors varied, depending on the source of the volatiles; each of the multiple pathogen-associated molecular patterns (PAMPs) used by microorganisms are recognized by different receptors, and they activate different pathways [6].

In conclusion, the present observations highlight the use of volatile organic components emitted from beneficial fungi as a new strategy for biocontrol. Although a volatile compound is difficult to apply in the field due to its evaporative nature and its efficacy is low compared with other chemical pesticides, some volatile compounds have been used successfully in the field to control plant disease [37]. On the other hand, chemical inducers of resistance are hampered by their own hazards including negative effects on plant growth [38]. MeBA and *m*-cresol have been used as antimicrobial compounds [39], and according to the material safety data sheet of Science lab.com, both (especially *m*-cresol according to Roberts et al. [40]) is corrosive to human skin and eyes at high concentrations. But in our observation, *m*-cresol was nontoxic to Arabidopsis plants even at high concentration (100 mM). Considering that point, both of these volatiles were able to prime systemic resistance even at very low concentrations, and perhaps only very low concentrations (1 µM) need to be applied (Fig. 4). However, further experiments in the greenhouse or open field using different crop plants are needed before these compounds can be recommended for commercial use.
